# Innovation in Informatics to Improve Clinical Care and Drug Accessibility for Rare Diseases in China

**DOI:** 10.3389/fphar.2021.719415

**Published:** 2021-10-15

**Authors:** Peng Liu, Mengchun Gong, Jie Li, Gareth Baynam, Weiguo Zhu, Yicheng Zhu, Limeng Chen, Weihong Gu, Shuyang Zhang

**Affiliations:** ^1^ State Key Laboratory of Complex Severe and Rare Diseases, Peking Union Medical College Hospital, Chinese Academy of Medical Science and Peking Union Medical College, Beijing, China; ^2^ Institute of Health Management, Southern Medical University, Guangzhou, China; ^3^ Digital Health China Technologies Co., LTD, Beijing, China; ^4^ Western Australian Register of Developmental Anomalies, King Edward Memorial Hospital, Perth, WA, Australia; ^5^ Division of Paediatrics and Telethon Kids Institute, Faculty of Health and Medical Sciences, Perth, WA, Australia

**Keywords:** rare diseases, health informatics, patient registry, cohort study, case reporting, digital health

## Abstract

**Background:** In China, there are severe unmet medical needs of people living with rare diseases. Relatedly, there is a dearth of data to inform rare diseases policy. This is historically partially due to the lack of informatics infrastructure, including standards and terminology, data sharing mechanisms and network; and concerns over patient privacy protection.

**Objective:** This study aims to introduce the progress of China's rare disease informatics platform and knowledgebase, and to discuss critical enablers of rare disease informatics innovation, including: data standardization; knowledgebase construction; national policy support; and multi-stakeholder participation.

**Methods:** A systemic national strategy, delivered through multi-stakeholder engagement, has been implemented to create and accelerate the informatics infrastructure to support rare diseases management. This includes a disease registry system, together with more than 80 hospitals, to perform comprehensive research information collection, including clinical, genomic and bio-sample data. And a case reporting system, with a network of 324 hospitals, covering all mainland Chinese provinces, to further support reporting of rare diseases data. International standards were incorporated, and privacy issues were addressed through HIPAA compliant rules.

**Results:** The National Rare Diseases Registry System of China (NRDRS) now covers 166 rare diseases and more than 63,000 registered patients. The National Rare Diseases Case Reporting System of China (NRDCRS) was primarily founded on the National Network of Rare Diseases (NNRD) of 324 hospitals and focused on real-time rare diseases case reporting; more than 400,000 cases have been reported. Based on the data available in the two systems, the National Center for Health Technology Assessment (HTA) of Orphan Medicinal Products (OMP) has been established and the expert consensus on HTA of OMP was produced. The largest knowledgebase for rare disease in Chinese has also been developed.

**Conclusion:** A national strategy and the coordinating mechanism is the key to success in the improvement of Chinese rare disease clinical care and drug accessibility. Application of innovative informatics solutions can help accelerate the process, improve quality and increase efficiency.

## Introduction

Rare Diseases refer to diseases with a very low incidence, often chronic and progressive, and life-threatening (Orphanet, 2021). The rare disease database Orphanet already contains 6,172 rare diseases, of which 71.9% are genetic and 69.9% are exclusively pediatric onset (Nguengang Wakap et al., 2020). Patients with rare diseases generally need long-term or even lifelong treatment, which seriously affects the quality of life of patients. And because of the high cost of treatment, it has brought a great economic burden to individuals, families, and society.

There is no single, widely accepted definition for rare diseases. Three elements to the definition as used in various countries are as follows: the total number of people having the disease, its prevalence, non-availability of treatment for the disorder (Richter et al., 2015). In the United States, a rare disease is defined as a condition that affects fewer than 200,000 people in the US (The Genetic and Rare Diseases Information Center, 2021). The European Union considers a disease as rare when it affects less than 1 in 2,000 citizens (EURORDIS-Rare Diseases Europe, 2021). In Korea, rare diseases are defined as diseases that affect fewer than 20,000 people or diseases for which an appropriate treatment or alternative medicine has yet to be developed (Song et al., 2012). In Australia, a disease is considered rare if it affects less than 5 in 10,000 people (Australian Government Department of Health, 2020). In China, rare diseases have not been officially defined. In 2018, the Chinese government officially released its first list of rare diseases, which included 121 rare diseases (The National Health Commission, 2019). The list has served as a reference for relevant government agencies and ministries.

Rare diseases are a global public health challenge. In China, people living with rare diseases have severe unmet needs. Health for All is China’s national healthcare strategy and the improvement of clinical care and drug accessibility for rare disease patients is a key issue to meeting its target. Epidemiological and clinical data for most rare diseases, which provide a foundation for policymaking at both the regional and national level, are missing in China (He et al., 2019). This is partially due to the lack of informatics infrastructure, including standards and terminology, data sharing mechanisms and networks, and concerns over patient privacy protection.

The Healthy China 2030 Planning Outline was issued by the Communist Party of China (CPC) Central Party Committee and the State Council in 2016 (The State Council, 2016). The Outline states that national health is the fundamental purpose of building a healthy China. Since there are about 20 million patients who suffer from rare diseases in China (Lane, 2019) improving clinical care and drug accessibility for people living with rare diseases is of great significance to addressing the elements of the Outline. Furthermore, people living with rare diseases are frequently vulnerable (1). So in addition to the very significant numerical arguments to prioritize the public health importance of rare diseases, the principle of social equity is also at the forefront.

## Methods

**FIGURE 1:**
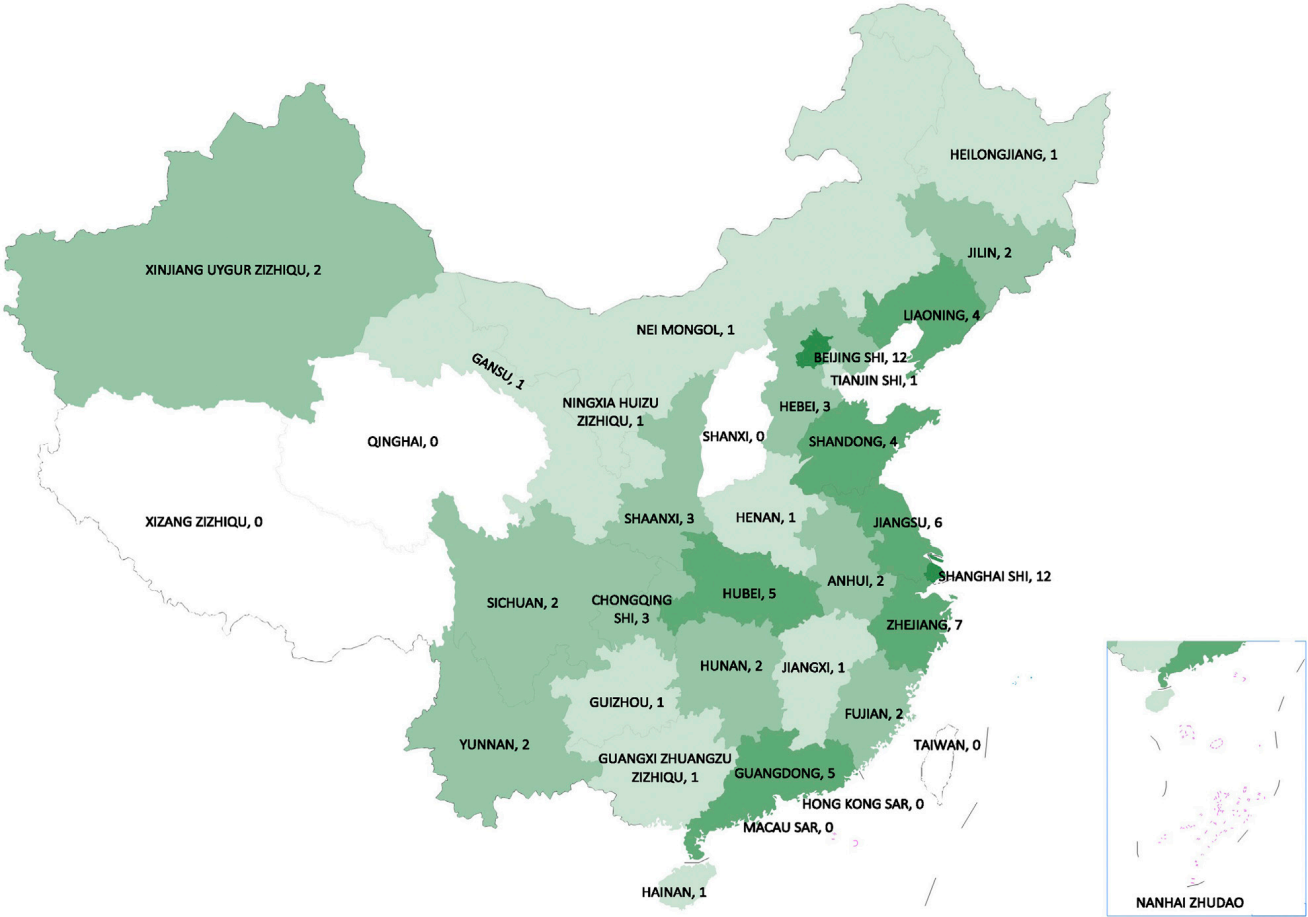
The regional distribution map of registered hospitals from NRDRS (as of Mar. 24, 2021). (The National Rare Diseases Registry System of China, 2021).

A systemic national strategy has been implemented to build the rare diseases informatics infrastructure to inform and support patient management. With funding from the Ministry of Science and Technology, the Peking Union Medical College Hospital (PUMCH) has developed the National Rare Diseases Registry System (NRDRS, www.nrdrs.org.cn) (Feng et al., 2018). An initial collaboration of more than 20 hospitals, which has grown to 88 (as of Mar. 24, 2021) (Figure 1). The aim is to compile a comprehensive research database that includes clinical, genomic, and bio-sample data and to support cohort studies to ultimately transform rare diseases care.

Meanwhile, with the endorsement of the National Health Commission of China, the PUMCH serves as the national rare diseases center, which is mainly responsible for taking the lead in establishing and improving the working mechanism of the collaboration network, formulating a national plan for doctor training, and treating the most critically ill patients with rare diseases, provides planning, coordinating, management, and technical support, and coordination of a national network of 324 hospitals for conducting direct case reporting on the 121 rare diseases included in China’s First List of Rare Diseases. An online registry updated manually and available to all member hospitals, is complemented by automatic reporting by the member hospitals to integrate their electronic medical record systems with the case reporting server provided by the national center. With the coverage of 324 medical centers designated as the national (1), and regional (323) rare disease centers, the statistician inside each center report weekly all the diagnosed cases of the rare diseases included in the first list of rare diseases. The annual report of the data will be published by the national rare disease center.

International standards, including Human Phenotype Ontology (HPO) and Logistic Observation Identifiers Names and Codes (LOINC) were applied to ensure interoperability and to support future multi-national studies. Privacy issues were addressed through HIPAA compliant rules. To ensure alignment to the needs of the rare diseases community, increase coverage and population representativeness, a multi-stakeholder involvement strategy was incorporated.

## Results

**FIGURE 2:**
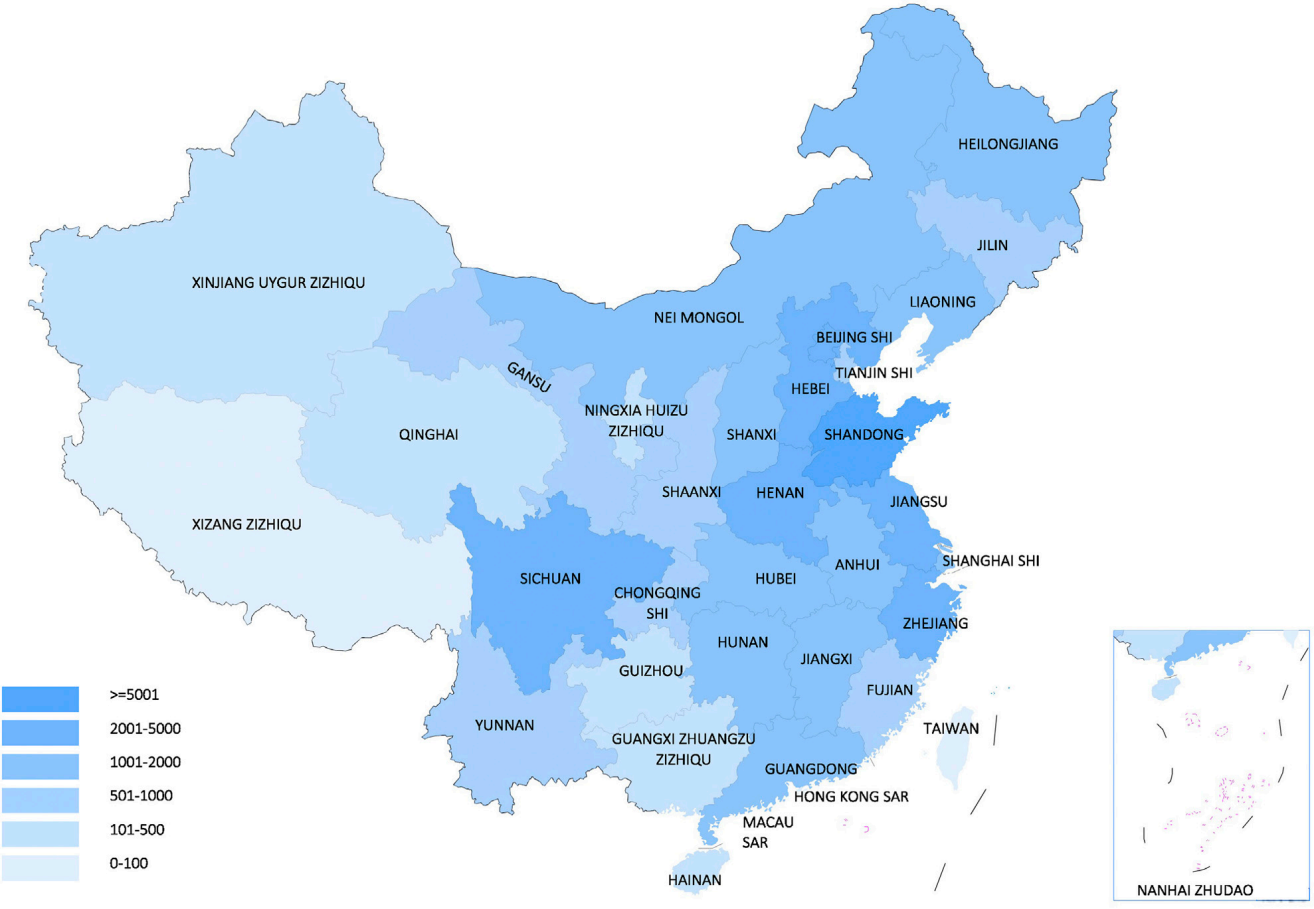
The regional distribution map of registered patients from NRDRS (as of Mar. 24, 2021). (The National Rare Diseases Registry System of China, 2021).

The NRDRS now covers the registry of 166 rare diseases and 63,470 registered patients in 185 case reporting forms (CRF) (as of Mar. 24, 2021) (Figure 2). A regional distribution map of registered patients has been launched and can be updated in real time (The National Rare Diseases Registry System of China, 2021). Both structured and unstructured data are collected in the NRDRS. Due to different research purposes and disease specificities, the number of items in each CRF varies greatly, with a median of 90. For the NRDRS, there are two ways to input the data. Firstly, the researchers can input the data manually on the website. Secondly, for the existing databases managed by different researchers, an Extraction-Transformation-Loading process and the data quality assurance process will be performed. Currently, the diseases registered have been partially connected to the Chinese Human Phenotype Ontology Consortium (CHPO) and LOINC. A series of system procedures have been established, including for: application approval, CRF approval, data export, to ensure principal investigators’ professionalism and authority in the field of rare disease research, diagnosis, and treatment and high data quality and security. Elements of the regular data quality assessment includes data integrity measures, validity checks, and repeated registration, to promote the continuous improvement of data quality and platform functions. All staff exposed to patient data in the NRDRS have HIPAA certification and receive annual HIPAA training. Patients are informed when they received healthcare services in clinical sites by the providers. Some of them signed the consent form specifically designed for the NRDRS. Some signed the general consent form provided by the hospitals. If the patient refused to sign the consent, the providers will not input their data into the NRDRS. The NRDRS also provides a platform for the establishment of a multicenter rare disease research group. Researchers from different hospitals who study the same disease are able to sign a group agreement to collaborate online and share their data according to the rules of data sharing within the group (Guo Jian and Li, 2021). The establishment of research groups has facilitated the collection and sharing of data and helped bring together experts in the same disease field. The involvement of a diverse range of rare diseases and researchers allows the system to build a shared, cross-linking system, which makes collaborative disease research possible and surfaces knowledge across disease domains. The institutes included in the project play a leading role in the research, diagnosis, and treatment of rare diseases in each province. As a national information platform, the NRDRS has standardized system procedures, strict control of data quality and security, and can provide opportunities for multi-center research cooperation, which makes the number of hospitals and patients registered on the NRDRS continue to increase.

In February 2019, 324 hospitals, representing all the provinces in mainland Chinese provinces, were selected based on their capacity and experience in treating patients with rare diseases to form the National Network of Rare Diseases (NNRD) (The National Health Commission, 2019a). This collaborative network is of great significance for two-way referrals, Medical professional training, drug availability, clinical research, and case reporting of rare diseases. In November 2019, the National Rare Diseases case Reporting System of China (NRDCRS) was officially launched, mainly based on the collaborative network of these 324 hospitals and focused to real-time case reporting (The National Health Commission, 2019b9). The NRDCRS now has 324 member hospitals, and more than 400,000 cases have been reported to the national center (as of Sept. 1, 2020). On the NRDCRS, all rare diseases use the same report form, which has less than 60 items. It collects data on personal details, diagnosis and treatment, family history related to the disease, medical insurance type, medical costs, personal and family income, and follow-ups of patients with rare diseases. The data is hosted in the National Key Laboratory for Rare Diseases and Critical Care of China. The NRDCRS is a database that’s connected to the hospital information system directly, in some hospitals, or manual input in some others. The central servers are both physically in the national key lab. For DPRSRD, the main way to input the data is case-by-case collection of the data inside each regional center, following a standard form, and weekly submission of the data collection form to the national center. In the national center and some regional centers, this form can be generated by the electronic medical records system automatically. The EMR system used by these hospitals shows a report form that’s specially designed for the patients with rare diseases. In contrast to the NRDRS, the NRDCRS is a national-level policy that is enforced nationwide. The account registration of the NRDCRS is only open to designated hospital reporters, hospital administrators, provincial administrators, and national administrators, and the hospital administrator needs to review the quality of the data entered by the hospital reporter. The establishment of the NRDCRS to collect relevant data is conducive to the understanding of the current status of rare diseases’ epidemiology, clinical diagnosis and treatment, and medical security in China. It provides a scientific basis for formulating crowd intervention strategies, improving the diagnosis and treatment services system, the level of patient medical security, and drug accessibility.

Based on the data and real-world evidence available in the two systems, the National Center for Health Technology Assessment (HTA) of Orphan Medicinal Products (OMP) has been established. Members of the center are widely representative, including rare disease research experts, HTA research experts, policymakers, clinical therapists, representatives of pharmaceutical companies, payers, and patients’ rights and other stakeholders. And the expert consensus of HTA on OMP has been produced and published (China Alliance for Rare Diseases, 2019). This serves as the technical guide for the industry to perform safety, efficacy, and economic assessments of OMP and provides evidence for OMP market access approval and insurance coverage. In China, market access to OMP, and the progress in medical insurance reimbursement are still unable to meet the growing demand. For licensed OMP, the challenge lies in the lack of standardization in the evaluation of the effectiveness, safety, economy, and other aspects of OMP; therefore orphan-drug pricing and medical insurance reimbursement lack a fully developed policy basis. On the other hand, due to the characteristics of rarity and heterogeneity of rare diseases, more valid information—such as the prevalence or incidence standard of rare diseases—is necessary, especially during the early research and development stage (Nestler-Parr et al., 2018), but this information is often lacking. The development of the NRDRS and NRDCRS can serve as a reliable data ecosystem for providing the epidemiological information, diagnosis and treatment information of rare diseases, which will thereafter support the early registration of drugs and real-world evidence studies for rare diseases in China. This information could help establish an accurate estimation of benefit of new OMP in relation to costs (Pearson et al., 2018).

The largest knowledgebase for rare diseases in Chinese has also been produced, including *the Diagnosis and Treatment Guidelines for 121 Rare Diseases* (The National Health Commission, 2019c), *Compendium of China’s First List of Rare Diseases* (The Peking Union Medical College Hospital, 2018), and the translation of the HPO and GeneReviews into Chinese (The Chinese Human Phenotype Ontology Consortium, 2021), (The GeneReviews Chinese Version, 2021). The CHPO project was launched in December 2015, with more than 180 professional participants. Wiki websites and search engines have been established and are continuously optimized. The CHPO connects the Online Mendelian Inheritance in Man (OMIM) database and translates the OMIM disease directory, and maintains close cooperation with the HPO team to keep the thesaurus up to date. The CHPO has established an audit committee, which is responsible for the editing and optimization of Chinese translations and definitions of different categories of vocabulary. As of October 2016, after removing duplicate entries in the different classifications, the total number of entries reached 11,896. More than 50 institutions and project teams have applied to download the CHPO thesaurus, including multiple genetic testing institutions, hospitals, universities, and research institutions (CHPO wiki, 2021). The GeneReviews (National Library of Medicine, 2021) translation project was launched in December 2016 to connect relevant professionals to translate and publish GeneReviews. As a part of China’s rare disease knowledge base, it provides professional support for domestic genetic and rare disease diagnosis and treatment, and genetic consultation. As of August 29, 2019, 279 items have been claimed. After translation and review, 264 items have been uploaded and 70 claimants/teams verified. Website visits have increased steadily (The GeneReviews Chinese Version, 2021). Collectively, these knowledgebases provide an additional basis for continued medical education for healthcare service providers working with rare diseases.

## Discussion

Difficulties in the diagnosis and treatment of rare diseases and high rates of misdiagnoses and missed diagnoses are common in China (Dong et al., 2020). In addition, rare disease medicine accessibility and affordability issues are prominent, and a data basis for policy formulation is lacking. For instance, the lack of epidemiological survey data makes it challenging to arrive at a unanimous definition of rare diseases, and the development and implementation of many rare disease policies were based only on China’s First List of Rare Diseases released in 2018. However, the national coordinating strategy (Table 1) for the management of rare diseases is successful in promoting the accessibility and quality of clinical care for rare diseases patients in China. Specifically, it: ① *Encourages enterprises to develop treatment for rare diseases*. Due to a series of preferential policies implemented by the Chinese government for rare diseases (The Central People’s Government of the Peoples Republic of China, 2021), pharmaceutical companies have increased their enthusiasm for the development of rare disease drugs. Simultaneously, the implementation of a registration system and a direct reporting system has made it easier to recruit patients for clinical trials of rare disease drugs. ② *Improves the ability of medical staff to diagnose and treat rare diseases*. The NNRD was established to support the training of clinical physicians specialized in rare diseases, improve their ability to diagnose and treat rare diseases, speed up the time required to confirm a disease diagnosis, and reduce the rate of misdiagnoses and missed diagnoses. ③ *Provides a data basis for rare disease policy formulation*. The nationwide, compulsory implementation of NRDCRS provides epidemiological evidence, such as the number of rare disease patients, prevalence rate, incidence rate, and geographical distribution. In addition, this direct reporting system and the hospital medical insurance system in China also collect data on rare disease medical costs. Collectively, these data provide a knowledge basis for the Chinese government to formulate rare disease policies. This can serve as a model that is adaptable for other countries, especially those with large populations.

**TABLE 1:** The national coordinating strategy for the management of rare diseases.

Outcome	Improving the accessibility of health services for rare diseases										
↑											
**Process**	Multicenter research groups	Rare diseases epidemiology		Medical professional training		Patients referral and hierarchical medical system			Expert consensus on HTA of OMP		
↑											
**Structure**	Informatics infrastructure			Government-led			Multi-stakeholder involvement				
	NRDRS	NRDCRS	Other rare disease information system (e.g. DPRSRD)	Scientific research projects	Policy making (e.g. NNRD)	Direct reporting of rare diseases	Government	Hospitals	Research institutions	Patients	Medical industries
	Knowledgebase construction										
	Patient privacy protection										
	Links to international terms										

Standardization and analytics of data from different sources is difficult and implementation of the international standards is key. The development of wearable devices, cloud storage, artificial intelligence (AI), genetic sequencing, and other technologies are enabling for the collection, storage, transmission, and analysis of health data. Biomedical and clinical data are being generated by the terabyte, and even petabyte. The importance of the application of real-world evidence in medical decision-making is gaining increasing acceptance, especially for rare diseases (Food and Drug Administration, 2018; National Medical Products Administration, 2020). Worldwide data sharing and international collaboration are increasingly promoted. However, most patient data flow from heterogeneous systems for different purposes using different software, file formats, and data models (Basu et al., 2019). This has increased the demand for data standardization and quality management. Data standardization—the process of transforming data into a common format that can be understood across different tools and methodologies (He et al., 2019)—has attracted extensive attention and led to many related studies (Basu et al., 2019), (Park et al., 2019). Common data models (CDMs) are a mechanism by which raw data are standardized to a common structure, format, and terminology independent of any particular study (Cohen et al., 2020), as well as rare diseases registry (RD-Connect Project, 2021). The implementation of the international standards is helpful, such as SNOMED CT, LOINC, Orphacodes, Medical Dictionary for Regulatory Activities (MedDRA), and Unified Medical Language System (UMLS).

Phenotypic information, which is key to the accurate diagnosis of rare diseases (Baynam et al., 2015), is not routinely collected as coded or standardized terms (Mooney and Pejaver, 2018). Thus, researchers could develop their own forms for patient registration that contain many created or localized terms and, in turn, can promote the creation of localized terms (Sloboda et al., 2018). In particular, patient information differs significantly within the global community. The translation and promotion of the CHPO is helping ensure fluent culturally appropriate communication within the global community and solve related problems. The application of natural language processing technologies to extract phenotypic information from electronic medical records and terms in registry systems facilitates global communication. Besides, with the development of image extraction technology, facial image collection and analysis is also a potential method to increase diagnostic proficiency in hospitals (Hurst, 2018; Baynam et al., 2017; CLINIFACE, 2021). Facial recognition is one phenotyping technology that is increasingly being used to support rare disease diagnosis, many rare diseases have a characteristic, but often subtle, facial phenotype. Moreover, facial abnormality is likely to be unappreciated by physicians, at least by physicians with less experience. Using computer assistance will help solve this problem (Hadj-Rabia et al., 2017), (Basel-Vanagaite et al., 2016).

Solid and sustainable funding from the central government is key to the success of this registration and case reporting system. The initial funding from the Ministry of Science and Technology of China was an important stimulus for research on rare diseases (The National Science and Technology Information system, 2016). Multi-stakeholder involvement can help build a sustainable ecosystem to support long-term development that serves the needs of the rare diseases community. Patient advocacy groups (PAGs), also called patient advocacy organizations (PAOs), are of great importance in the clinical care and research of rare diseases (Koay and Sharp, 2013; Merkel et al., 2016; House et al., 2019). PAGs can be an important source of diagnostic, treatment, and follow-up data. For example, the 2019 Comprehensive Social Survey of Rare disease Patients in China, led by the China Alliance of Rare Diseases (CHARD), and implemented by the Jockey Club School of Public Health and Primary Care of the Chinese University of Hong Kong (CUHK), successfully interviewed rare disease patients scattered across China with the help of PAGs, and conducted a high-quality survey (China Alliance for Rare Diseases, 2019). Additionally, a survey of the Rare Diseases Clinical Research Network (RDCRN) also showed that PAGs could play an important role to recruit patients for RDCRN studies (Merkel et al., 2016). After the NRDCRS went online in 2019, CHARD launched the Direct Patient Reporting System of Rare Diseases (DPRSRD). As an important supplement to the NRDCRS, the DPRSRD uses a form consistent with that of the NRDCRS, to collect medical and social information on rare diseases patients. Patients or their family members can report disease information on the WeChat terminal of their mobile phone. The DPRSRD has broken through the time and geographical limitations of case reporting, and has made it easier to collect certain information, such as indirect costs and family economic status. It shows, under the strong advocacy of the PAGs, that the patients are highly motivated to report the case information and the data are relatively complete. This is encouraging, however, the accuracy of patient and family-reported data needs further consideration. The false-positive rate and the consistency of reporting between patients and doctors (Muggah et al., 2013) needs to be determined, including by comparing the data of NRDCRS and DPRSRD in future studies. The transfer of data through social media platforms such as WeChat also raises concerns over cybersecurity, privacy breaches, and discrimination that could also be assessed in further studies. As the new regulation on protection of personal information in China (The Central People’s Government of the People’s Republic of China, 2021), an information collection system directly connected with the patients, such as DPRSRD, met unique challenges, including consent, technologies to ensure the withdraw of the submitted information and so on. Finally, accommodating multiple Chinese languages, including Indigenous languages, will be important for culturally appropriate, diverse and equitable approaches (PROJECTY, 2021) to improving the lives of people living with rare diseases. The differences between NRDRS, NRDCRS, and DPRSRD are shown in Table 2.

**TABLE 2:** The differences between **NRDRS, NRDCRS and DPRSRD**.

Differences	National rare diseases registry system of China (NRDRS)	National rare diseases case reporting system of China (NRDCRS)	Direct patient reporting system of rare diseases (DPRSRD)
System builder	Peking Union Medical College Hospital (PUMCH)	National Health Commission	China Alliance for Rare Diseases (CHARD)
Launch time	July 2017	November 2019	November 2019
Construction background	Relies on the National Key Research and Development Program of China (2016YFC0901500) and was established primarily for cohort studies of rare diseases	Collects information on patients with rare diseases based on the NNRD.	It supplements the NRDCRS, and used the ability of PAGs to identify patients with rare diseases and collect information on each patient as completely as possible
Main purposes	1. Built for the “Rare disease Clinical Cohort Study” project. It is a registration platform for patients with rare diseases	Provides medical staff with direct case histories on the reporting of rare diseases	1. Provides rare disease patients and their families with a tool to report on rare diseases
	2. Provides the public with information to increase their knowledge of rare diseases and share some of the data on rare cases		2. Supplements the NRDCRS to improve the data integrity of patients with rare diseases
Primary objectives	1. To establish unified technical standards and norms for the registration of rare diseases	1. To obtain epidemiological information on rare diseases and to assist in formulating a definition of rare diseases	To collect comprehensive information on each rare disease patient
	2. To form a national rare disease research cooperation network by combining top-level units	2. To establish a patient address book to facilitate a connection between diagnosis and treatment needs and clinical trials	
	3. To carry out the registration and research of rare diseases nationwide	3. To support the establishment of a standard diagnosis process and the development of diagnosis and treatment guidelines, and clinical pathways	
	4. To promote the clinical diagnosis and treatment ability of rare diseases in China	4. To support the decision-making of the health commission and the medical insurance management department	
Users	Public and qualified clinical researchers	Designated hospital reporters, hospital administrators, provincial administrators, and national administrators	Patients with rare diseases and their families
Rare diseases	Multiple diseases	Multiple diseases	Multiple diseases
Number of forms used for data collection	Multiple sets of data collection forms are provided by the creators of each rare disease cohort. Researchers can design different disease forms according to different research needs	All rare diseases share a set of data collection forms to collect basic information, general conditions, diagnosis and treatment information, disease family history, diagnosis and treatment costs, medical records, and examinations of patients with rare diseases	All rare diseases share a set of data collection forms to collect data. The field settings are almost the same as those of NRDCRS, except that data upload is not supported

## Conclusion

A national strategy and coordinating mechanism is key for improvement of clinical care and drug accessibility in the treatment of rare diseases in a country with a large population. The application of innovative informatics solutions can help accelerate the process, improve quality, and increase efficiency. With the registry system for scientific research, case reporting system for public health service and policymaking, direct patient and family data ascertainment and the related data-driven systems and services, China has built a data infrastructure for rare diseases research and management to address the unmet medical needs of patients with rare diseases and to achieve the national goal of Health for All.

## Abbreviations

CHARD, China Alliance of Rare Diseases; CHPO, The Chinese Human Phenotype Ontology Consortium; CRF, case reporting form; CUHK, Jockey Club School of Public Health and Primary Care of the Chinese University of Hong Kong; DPRSRD, Direct Patient Reporting System of Rare Diseases; HPO, Human Phenotype Ontology; HTA, Health Technology Assessment; LOINC, Logistic Observation Identifiers Names and Codes; NNRD, National Network of Rare Diseases; NRDCRS, National Rare Diseases Case Reporting System of China; NRDRS, National Rare Diseases Registry System of China; OMIM, Online Mendelian Inheritance in Man; OMP, orphan medicinal products; PAGs, patient advocacy groups; PAOs, patient advocacy organizations; PUMCH, Peking Union Medical College Hospital; RDCRN, Rare Diseases Clinical Research Network.
